# CMPK1 Regulated by miR-130b Attenuates Response to 5-FU Treatment in Gastric Cancer

**DOI:** 10.3389/fonc.2021.637470

**Published:** 2021-03-18

**Authors:** Huaizhu Chu, Nahui Han, Jianguo Xu

**Affiliations:** ^1^ Department of Oncological Surgery, Qinghai Provincial People’s Hospital, Xining, China; ^2^ Department of Pain Medicine, Qinghai Provincial People’s Hospital, Xining, China

**Keywords:** MiR-130b, CMPK, 5-FU, chemoresistance, gastric cancer

## Abstract

Gastric cancer (GC) remains a major world-wide challenge, especially in Asian countries. Chemotherapy with 5-fluorouracil (5-FU) and cisplatin is used as the first-line treatment and development of chemoresistance is a major cause of progression. UMP/CMP kinase is responsible for the phosphorylation of the ribonucleotide metabolite 5-fluoro-5′-monophosphate (FUMP) in 5-FU metabolic process, and recognized as a key step in the conversion of 5-FU to cytotoxic metabolites. Our bioinformatics analysis and molecular experiments demonstrated that high expression of CMPK1 was associated with prolonged survival and response to 5-FU treatment in GC samples. Further analysis demonstrated that miR-130b as a key epigenetic regulator of CMPK1, and miR-130b-mediated attenuation of CMPK1 resulted in resistance of gastric cancer cells to DNA damage and cell death after treatment with 5-FU. Rescue experiments with augmented CMPK1 expression abolished the effect of miR-130b demonstrating the key function of this miRNA in this pathway. Thus, this newly identified miR-130b-CMPK1 axis suggests a potentially new chemotherapeutic strategy for improved response to 5-FU therapy.

## Introduction

Gastric cancer (GC) is a leading cause of cancer-related death worldwide (1). Although clinical outcomes of GC have gradually improved through surgical resection and chemotherapy in China, 5-year survival rates of patients with GC are only 20%-30% (2). Generally, fluoropyrimidine (i.e., 5-fluorouracil (5-FU), capecitabine, or S-1)- and platinum (i.e., cisplatin or oxaliplatin)- based regimens are recommended as the primary treatment regimen (3, 4). Acquired resistance to 5-FU or platinum is considered the major cause for disease progression (5, 6). Previous studies revealed the 5-FU metabolism activation-related enzyme, including thymidylate synthase (*TYMS)*, orotate phosphoribosyltransferase (*OPRT)* and dihydropy-rimidine dehydrogenase (*DPYD*) were involved in 5-FU sensitive and recognized as response biomarkers of tumor chemosensitivity, whereas the prediction efficacy remains unsatisfactory (7, 8). Identifying new targets involved in drug resistance may help oncologists choose more appropriate chemotherapy for improved survival.

Human UMP/CMP kinase (CMPK1) is responsible for metabolism of CMP, UMP, and deoxycytidine analogs, many of which are important anticancer and antiviral agents, as well as fluoropyrimidines (FPs) (9, 10). Nucleoside analogs need to be phosphorylated stepwise to their triphosphate forms to exert their full therapeutic effect (11). The main mechanism of 5-FU activation is conversion to 5-fluorouridine 5′-monophosphate (FUMP), with subsequent phosphorylation to the corresponding diphosphate (FUDP) and triphosphate (FUTP) forms. While FUTP may be incorporated into RNA and disrupt diverse cellular processes, FUDP may be converted by ribonucleotide reductase to 5-fluoro-2′-deoxyuridine 5′-diphosphate (FdUDP), which can either be phosphorylated or dephosphorylated to generate the active deoxyribonucleotide metabolites FdUTP and FdUMP, respectively. FdUMP inhibits thymidylate synthase while FdUTP is incorporated into DNA, leading to cellular damage (7). Studies have suggested that UMP/CMPK is responsible for the phosphorylation of FUMP to the diphosphate and triphosphate metabolites (11–14).

MicroRNAs (miRNAs) are small non-coding RNAs (20–25 nucleotides) that regulate expression of target proteins through degradation of mRNA or translational inhibition (15). Accumulating evidence shows that miRNAs are frequently dysregulated in many human cancers, including GC (16–19). Aberrant miRNA expression has been reported to be a contributing factor in multiple tumor physiological and pathological processes, including proliferation, invasion, apoptosis and chemotherapy resistance (20–23). Thus, miRNAs may function as diagnostic or prognostic markers. TCGA program (https://cancergenome.nih.gov/) have curated the detailed clinical annotation (including drug type and response status) and integrated miRNA and mRNA expression profiles (24), providing the abundant resource to identify the chemosensitivity biomarker. However, it is unknown how molecular alterations is involved in the chemotherapy response and the upstream regulatory targets are still not well characterized.

In the present study, we investigated mRNA and miRNA expression profiles in TCGA samples of received 5-FU-based therapy and identified CMPK1, a novel downstream gene target of miR-130b, was related to early progression and 5-FU sensitivity in patients with gastric cancer. Moreover, the siRNA-mediated repression of CMPK1 phenocopies all of the miR-130b mimics that triggered 5-FU sensitivity changes in vitro and in vivo experiments. This newly identified miR-130b-CMPK1 axis provided a potentially new chemotherapeutic strategy for improved response to 5-FU therapy of GC patients.

## Materials and Methods

### Samples and Clinical Data

The clinical-pathological information, microRNA and mRNA expression profiling for 68 gastric cancer samples received 5-FU-based therapy were curated from TCGA dataset and downloaded from the UCSC Xena (GDC hub: https://gdc.xenahubs.net). 5-FU-based chemotherapy were defined as patients who received 5-Flourouracil or Capecitabine or Doxifluridine or Xeloda regimens. GC patients in TCGA cohort were divided into response or non-response subtype based on the clinical benefits. The miRNA and mRNA microarray data for 25 tumor tissues were downloaded from GEO (ID: GSE36968) published by Kim et al. (25). Detailed clinical information, including age, gender, molecular subtype, response status, survival time, and specific gene expression, was also collected from aforementioned studies and is provided in **Supplementary Table S1**. The study was approved by the medical ethics committee at Qinghai Provincial People’s Hospital.

### Identification of DEGs Between Distinct 5-FU Clinical Response Subtype

The R package “limma” was used to evaluate DEGs in GC samples between different 5-FU clinical response subtype. Specifically, gene expression data were normalized by voom and then fed to lmFit and eBayes functions to calculate the differential expressed statistics. The significance filtering criteria of DEGs were set as an adjusted P value less than 0.05 and Fold change more than two times.

### GSEA and Network Analysis

Gene set enrichment analysis (GSEA) using R packages “ClusterProfiler” was utilized to evaluate differential expression of each gene in GC samples with different 5-FU sensitive subtype or CMPK1 expression subgroups. The differential expression statistics obtained from “limma” were used as input to perform GSEA based on KEGG gene set (download from MSigDB database v7.1). The fast gene set enrichment analysis algorithm was implemented in the ClusterProfiler and calculated with 10000 nperms. R package “enrichplot” was adopted to visualize GSEA result of CMPK1 high expression subgroup.

### Cell Culture and Transfection

Human gastric cancer cell lines (MGC-803, AGS) were purchased in 2015 from cell bank of CAS (Shanghai, China) which cultured in RPMI1640 (GIBCO, USA) medium containing 10% FBS and maintained at 37°C with 5% CO_2_. All cell lines were tested and authenticated by cell line typing analysis (STR profiling). The transfection of miR-130b mimic with miRNA control (miR-NC) (Ambion, USA) and CMPK1 siRNA with control siRNA (Shanghai GenePharma, China) were performed according to the manufacturer’s instruction using Lipofectamine RNAiMAX (Invitrogen, USA). The final concentration of miRNA and siRNA was 20 nM respectively.

### RNA Extraction Quantitative RT-PCR and Western Blot Analysis

Expression levels of miR-130b and CMPK1 mRNA in gastric cancer cell lines (MGC-803, AGS) were detected by qRT-PCR analysis and summarized in **Supplementary Methods**. Expression levels of CMPK1 (Abcam, US) and β-actin (Santa Cruz Biotechnology, US) proteins in GC cell lines were detected by western blot analysis. The brief description of western blot was also summarized in **Supplementary Methods**.

### Luciferase Assay

CMPK1 3′-UTR sequence was amplified from cDNA with the CMPK1 3′-UTR up primers SacI (5′-GAGCT′CGCTTCCTTTCATCAGGTATC-3′) and down primers XhoI (5′-CTCGAGCATCCAACATCACTGAATGG-3′). The PCR products were then subcloned to the pmirGLO dual-luciferase target expression vector (Promega, USA) as wild-type vector pmirCMPK1-3′-UTR-Wt (CMPK1-Wt). The mutant vector pmirCMPK1-3′-UTR-Mut (CMPK1-Mut) was obtained by site-directed mutagenesis using QuikChange^®^ Site-Directed Mutagenesis Kit (Stratagene, USA). AGS and MGC-803 were seeded in a 24-well culture plate in triplicate and were co-transfected with miR-130b mimic and miR-NC followed by CMPK1-Wt or CMPK1-Mut using DharmaFECT Duo Transfection Reagent (Thermo, USA) according to the manufacture’s procedure. Luciferase activity was normalized to that of pRL-TK luciferase. The cells were collected at 24h post-transfection; luciferase activity was measured by a dual-luciferase reporter assay kit (Promega, USA) and recorded by a GloMax 20/20 (Promega, USA).

### MTT Assay

Twenty-four h after transfection with 20 nm miR-130b, miR-NC, or miRNA-inhibitor, cells were seeded onto 96-well plates (3×10^3^ cells/well) and treated with a titration of 5-FU or cisplatin. After incubation for five days, cell viability was estimated using the MTT reagent (Solarbio, China), and surviving fractions were calculated. Cell survival was calculated by normalizing the absorbance to that of untreated controls.

### Colony-Formation Assay

Forty-eight h after transfection with 20nm miR-130b, miR-NC, or si-CMPK1, Cells were harvested. Transfected cells were seeded in a six-well plate (1000 cells/well) and allowed to grow for 3 to 4 days and then treated with 5-FU for 10 days, during which time the surviving cells spawned a colony of proliferating cells. Colony formation was quantified by staining the cells with 0.1% crystal violet and counting surviving colonies containing more than 50 cells.

### miRNA Target Prediction

TargetScan (http://www.targetscan.org/), PICTA (http://pictar.mdc-berlin.de/), miRDB (http://www.mirdb.org/), and miRTarBase (http://mirtarbase.cuhk.edu.cn/) were used to predict the candidate miRNAs may interact with target gene 3′-UTR. Venn diagram with R package “VennDiagram” was utilized to show the overlapped results of the four online prediction tools.

### Rescue Experiment

CMPK1 coding sequence (CDS) plasmid (without 3′-UTR) and the blank vector plasmid (vector-NC) (Genechem, China) were used in the rescue experiment. AGS cells were transfected with miR-130b mimic in the presence of vector-CMPK1 or vector-NC for 48 h by using Lipofectamine 3000 reagent (Invitrogen, USA) according to the manufacturer’s instructions. The expression level of CMPK1 was measured by western blot analysis.

### Comet Assay

Comet assays were performed per manufacturer’s instructions (Trevigen, Gaithersburg, MD). Proliferating AGS cells were transfected with miR-NC, miR-130b mimic and si-CMPK1. Twenty-four h later, transfected cells were treated with 20μg/ml 5-FU for 48 h, and then were analyzed by single-cell gel electrophoresis. Further details are described in the Supplementary Methods.

### Cell Cycle and Apoptosis Analysis

he cell cycle and apoptosis assay were analyzed by a flow cytometer (Guava™ easyCyte; Millipore, USA) to determine cell populations in different conditions according to the manufacturer’s protocol. The brief description of the cell cycle and apoptosis protocol was shown in Supplementary Methods.

### 3-D Culture

Cells were dissociated with trypsin and re-suspended in culture media. After transfer of the respective volume of cell suspension to a fresh tube, an appropriate amount of media was added (final cell concentration is 3000 cells/80μl). One-part matrigel was then mixed with one-part cell suspension and 160μl of above mixture was transferred to each well of a 48-well plate. Media (300 μl) was slowly added into each well of the plate, which was transferred to an incubator for several days. A further 300 μl culture media containing 5-FU or cisplatin was replaced on top of the growth layer. Cells were incubated at 37°C and 5% CO_2_ for 2 weeks and total colonies were counted.

### Immunohistochemistry Staining

Immunohistochemistry for CMPK1 (Abcam, ab225940), TUNEL (Millipore, S7100) and Cleaved Caspase-3 (Cell Signaling Technology, 9661) were performed using standard techniques according to manufacturer’s instructions. Sections were incubated with primary antibody overnight at 4°C and then were incubated with secondary antibody. The enzyme substrate was 3, 3-diaminobenzidine tetrahydrochloride (DAB).

### Statistical Analysis

All experiments were performed in triplicate. All statistical analyses were performed using the R Software for Windows (3.6.1) and GraphPad Prism 8.0 statistic software. The expression levels of miR-130b and CMPK1 were log2(-ΔΔct) transformed and analyzed as a continuous variable by means and standard deviations (mean ± SD). The correlation between the expression of miR-130b and CMPK1 in TCGA-STAD samples used the Pearson correlation test. Kaplan-Meier method was used for survival analysis, and the differences in survival were examined using the log-rank test with R package “Survival,” Associations between the expression of miR-130b and CMPK1 and GC survival were also examined with the Cox proportional hazards regression model at both univariate and multivariate levels. In expression and survival analysis, miR-130b and CMPK1 expression was usually categorized into high and low groups using the lower tertile value as a cutoff. The *in vitro* and *in vivo* experiments were analyzed by independent sample t-test or one-way ANOVA. All comparisons were two-sided with an alpha level of 0.05, and the Benjamini-Hochberg method was applied to control the false discovery rate for multiple hypothesis test.

## Results

### CMPK1 Expression, Response to 5-FU Treatment, and Survival in GC

**Figure 1:**
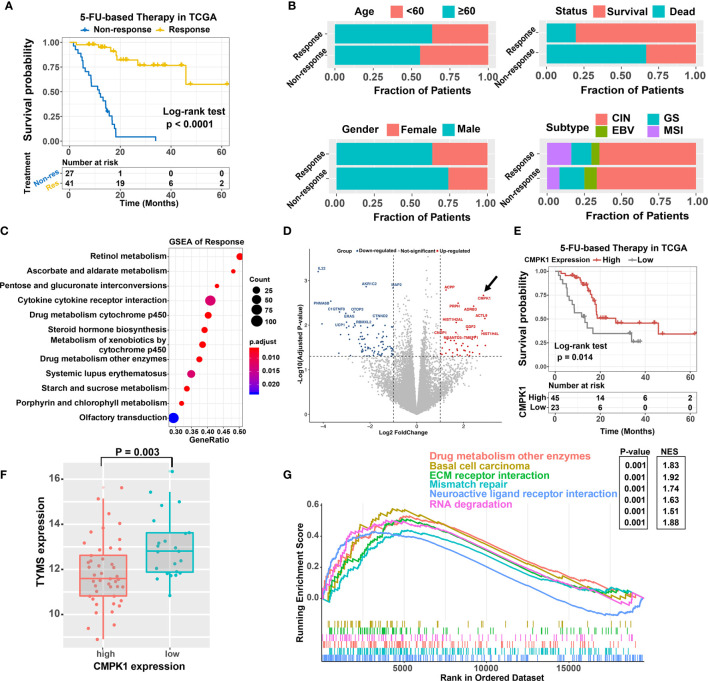
Identification of the CMPK1 affect the 5-FU chemosensitivity. **(A)** Kaplan-Meier survival curves of progression-free survival (PFS) for 68 GC patients with 5-FU-based-chemotherapy response (yellow line) or non-response (blue line). **(B)** Association of clinical features (age, gender, survival status, molecular subtype) and treatment response in GC samples. **(C)** Dysregulation of signaling pathways stratified by response versus non-response subtypes. **(D)** volcano plot of the differentially expressed genes in different treatment response subtypes. Blue and red dot indicated the genes highly expressed in non-response and response subgroups, respectively. **(E)** Kaplan-Meier survival analysis of PFS based on CMPK1 expression subgroups. The samples with upper two thirds CMPK1 expression were termed as high expression, the remaining were termed as low expression. **(F)** Distribution of TYMS in CMPK1 high versus low expression subgroup **(G)** Top enriched gene pathways in distinct CMPK1 expression subgroups (high vs low) were assessed by using the GSEA algorithm.

We first compared the clinicopathologic features between different 5-FU treatment response status in GC cohort. Patients with 5-FU sensitive subtype exhibited a significant prolonged progression-free survival (PFS) and overall survival (OS) time (PFS: HR = 0.06, 95% confidence interval (CI) = 0.02 to 0.16, P < 0.0001, **Figure 1A**; OS: HR = 0.22, 95% CI = 0.10 to 0.51, P = 0.0001, **Supplementary Figure S1A**). Besides, patients with older, female, and MSI subtype were more likely to be concentrated in the response subtype, although these differences were not statistically significant either (**Figure 1B** **)**. GSEA analysis revealed that biological processes involved in drug metabolism cytochrome P450, cytokine receptor interaction, ascorbate and aldarate metabolism et al. were markedly altered in GC samples with different 5-FU response subtype (**Figure 1C**). We next analyzed the differential expressed genes (DEGs) between the 5-FU response *vs* non-response subtype and identified several genes highly expressed in response subgroup (CMPK1, PRPH, ADRB3 et al.) (**Figure 1D** and **Supplementary Figure S1B**). Among these, the expression of CMPK1 was significantly associated with GC patients’ PFS and OS time (PFS: HR = 0.43, 95% CI = 0. 21 to 0.86, P = 0.014, **Figure 1E** **;** OS: HR = 0.48, 95% CI = 0.22 to 1.05, P = 0.058, **Supplementary Figure S1C**). A higher CMPK1 expression level was also observed in 5-FU response subgroup (**Supplementary Figure S1D**). Moreover, 5-FU chemotherapy resistance-related marker TYMS was highly expressed in CMPK1 low expression subgroup (**Figure 1F**). Signaling pathways involved in drug metabolism enzymes, ECM-receptor interaction et al. were significantly enriched in CMPK1 high expression subgroup (**Figure 1G** **).** Altogether, these results demonstrated that the expression of CMPK1 was involved in the regulation of 5-FU chemosensitivity in GC.

### CMPK1 Expression Impacts 5-FU Activation and the Cell Sensitivity in GC Cells

**Figure 2:**
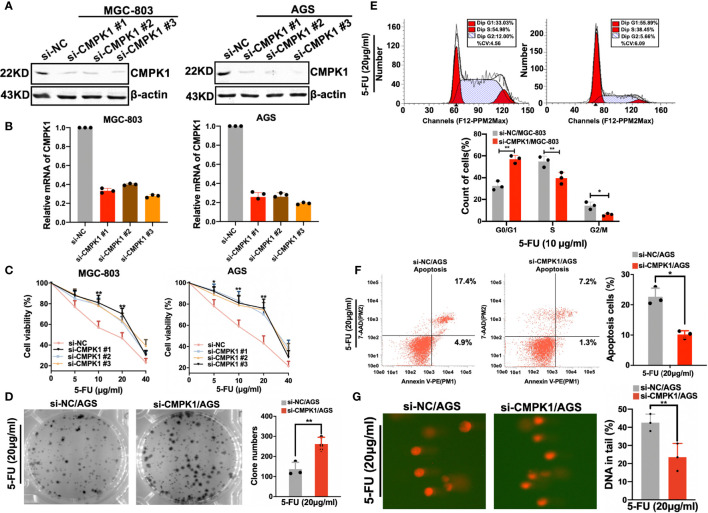
CMPK1 affects gastric cancer cell line sensitivity to 5-FU treatment. **(A, B)** AGS and MGC803 cells were transfected with 20 nM si-NC or si-CMPK1. After 48 h, cells were harvested for western-blot analysis **(A)** or RT-qPCR experiments **(B)**. **(C)** Cell viability of si-NC and si-CMPK1 with5-FU treatment was tested by MTT assay. **(D)** 5-FU sensitivity detection was re-confirmed in clonogenic cell-survival assay. **(E, F)** Apoptosis and cell-cycle analysis were pre-treated as previously described and measured by flow cytometry to determine the impact of CMPK1 treated with or without 5-FU. The representative flow cytometry patterns of cell cycle distribution and the statistical analysis was shown in **(E)**. The representative flow cytometry patterns of cell apoptosis and the statistical analysis was shown in **(F)**. **(G)** Knock-down of CMPK1 suppressed 5-FU-induced DNA damage determined by comet assay in AGS cells. Data are presented as mean ± SD of three independent experiments. *P < 0.05, **P < 0.01.

Since CMPK1 plays an important role in activation and cellular sensitivity to 5-FU, we wanted to confirm the effect in GC cells. Knockdown of CMPK1 *via* siRNA substantially reduced the protein expression and RNA level as compared to the internal reference control in MGC-803 and AGS cells (**Figures 2A, B** **)**. MTT assays indicated that CMPK1 suppression significantly reduced the GC cells response to 5-FU treatment (**Figure 2C** and **Supplementary Figures S2A, B**). As the three siRNA all exhibited excellent knock-down effect, we randomly selected si-CMPK1#3 as the following study target and named it si-CMPK1. Furthermore, a 2D clonogenic survival assay was implemented to confirm the sensitivity results (**Figure 2D**). As expected, CMPK1 knock-down in AGS cells resulted in resistance to 5-FU.

In cancer cells, 5-FU causes cell cycle arrest and impairs proliferation (26). We examined whether CMPK1 knockdown was capable of inhibiting 5-FU-induced cell cycle arrest in GC cells. Considering the AGS cell line was characterized by higher CMPK1 expression compared to MGC-803 cells, we selected it as the cell model in following experiments. As shown in (**Figure 2E**, upper panel), flow cytometry showed markedly reduced S-phase arrest in transfected si-CMPK1 cells than negative control (**Figure 2E**, lower panel).

Apoptosis was believed to be another primary mechanism responsible for 5-FU-induced cell death (27). To evaluate the 5-FU effect on cell apoptosis regulated by CMPK1, flow cytometry was used to assess cell status. We found that cells transfected with si-CMPK1 underwent less apoptosis after 5-FU treatment than cells transfected with si-NC, indicated that knockdown of CMPK1 reduced 5-FU-induced the apoptosis of GC cells (**Figure 2F** **).**

To determine whether CMPK1 downregulation affects 5-FU induced DNA damage, we measured the persistence of double-strand breaks as an indicator of damaged DNA (28). Single-cell gel electrophoresis (alkaline comet assay) was carried out to measure DNA damage. As shown in (**Figure 2G**, left panel), AGS cells with siRNA had lower levels of CMPK1 protein that lead to statistically significantly lower DNA damage than control cells (**Figure 2G**, right panel). The results indicated that GC cells treated with si-CMPK1 in combination with 5-FU had a significantly lower cytotoxicity.

### MiR-130b Directly Targets CMPK1 3′-UTR and Negatively Regulates its Expression in GC

**Figure 3:**
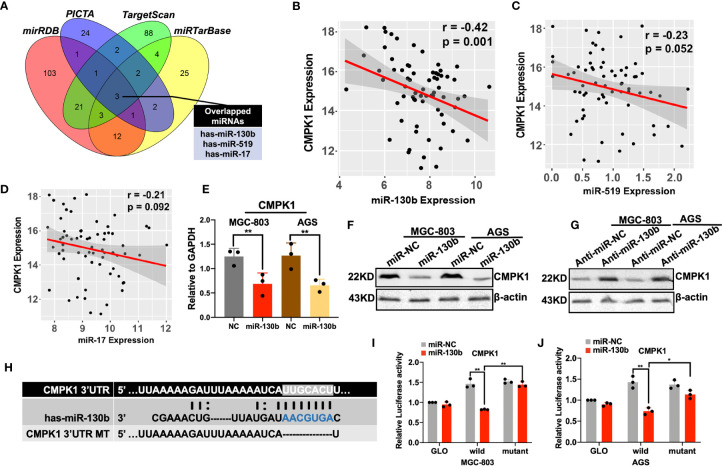
Prediction and validation of CMPK1 upstream regulator miR-130b in gastric cancer. **(A)** A Venn diagram showing the combination of four miRNA prediction algorithms identified three candidate upstream regulators of CMPK1. **(B–D)** Inverse correlation between CMPK1 and miR-130b, miR-519, and miR-17 in GC tissue **(E)** RT-qPCR analysis revealed that transfection with miR-130b mimic decreased the CMPK1 RNA level. **(F)**The protein level of CMPK1 was decreased in MGC-803 and AGS cells when transfected with miR-130b with β-actin as a loading control. **(G)** On the contrary, cells transfected with anti-miR-130b expressed increased levels of CMPK1 protein compared to those transfected with anti-miR-NC. **(H)** A putative miR-130b -binding site exists in the 3′-UTR of the CMPK1 mRNA, and 7-nucleotide deletion were generated in the binding site. **(I)** MGC-803 and **(J)** AGS cells transfected with pmirGLO-CMPK1-Wild and pmirGLO-CMPK1-Mut reporters, together with a miR-130b mimic or negative control, miR-130b overexpression suppressed the activity of luciferase in the wild-type but not in mutant type. All the values shown were represented as means ± SD. (*P < 0.05; **P < 0.01 *vs* negative control by two-tailed Student’s t-test).

To identify the upstream regulator that potentially modified the CMPK1 expression, we combined four public miRNA databases (miRDB, PICTA, TargetScan, and miRTarBase) and identified several putative miRNA binding sites in the CMPK1 3′-UTR region, including miR-130b, miR-519 and miR-17 (**Figure 3A** and **Supplementary Figure S3A** **).** We also analyzed the expression correlation between potential miRNAs and CMPK1, and identified the miR-130b were significantly negatively correlated with the CMPK1 in GC samples (**Figures 3B–D** **).** The negative correlation between CMPK1 and miR-130b was further corroborated in an independent GC cohort (GSE36968, **Supplementary Figure S3B** **)**. In addition, overexpression of miR-130b in the MGC-803 and AGS cells resulted in a significant reduction in CMPK1 mRNA transcription as well as protein expression by RT-qPCR and western blot assays (**Figures 3E, F** **)**. Conversely, we found that transfecting with miR-130b inhibitor (anti-miR-130b) resulted in an up-regulation of CMPK1 expression in the two GC cell lines (**Figure 3G**). Similarly, high expression of miR-130b in patients who received a 5-FU-based therapeutic regimen were more likely to develop malignant progression (**Supplementary Figure S3C**). Intriguingly, miR-130b expression was also significantly positively correlated with chemoresistance biomarker of TYMS, suggested its predictive value on chemotherapy efficacy (**Supplementary Figure S3D** **).** To further identify the functional interaction between CMPK1 and miRNAs generated from the prediction algorithms, we performed luciferase reporter assay by inserting the gene’s 3′-UTR region (wild type) or mutant CMPK1 3′-UTR vector (mut type) into downstream of the firefly luciferase plasmid (**Figure 3H**). In GC cell lines transfected with the wild type CMPK1-3′UTR and the miR-130b mimic, a significant decrease in luciferase activity was observed compared with mutant type CMPK1-3′UTR vector and mimic/NC controls (**Figures 3I, J** **)**. These results suggest that miR-130b negatively regulates CMPK1 gene expression.

### Effect of miR-130b on Sensitivity to 5-FU in GC Cells

**Figure 4:**
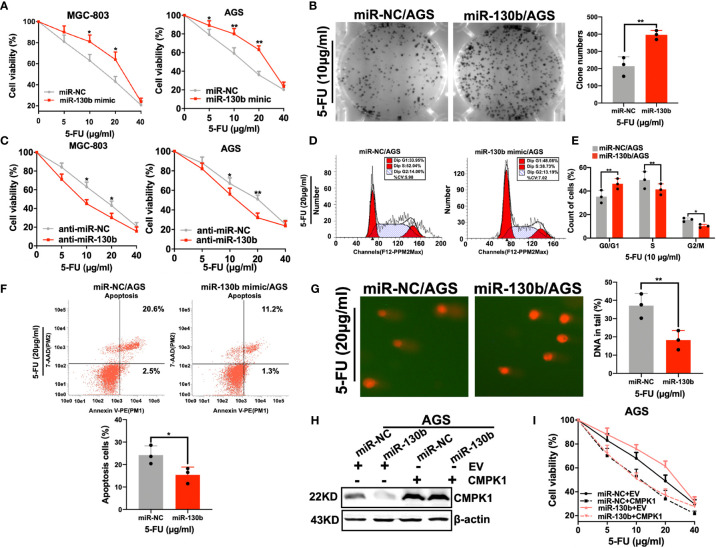
MiR-130b has an influence on 5-FU sensitivity. **(A, B)** AGS and MGC-803 cells were transfected with either miR-NC or miR-130b mimic for 48h, and cells were reseeded for 5-FU sensitivity detection using an MTT assay **(A)** and a clonogenic cell-survival assay **(B)**. **(C)** AGS and MGC-803 cells were transfected with either anti-miR-NC or anti-miR-130b upon 5-FU treated. Cell viability was assessed by MTT assay. **(D–F)** Cells in the apoptosis and cell-cycle analysis were pre-treated and measured by flow cytometry. AGS cells were used to determine the impact of miR-130b/control treated with 5-FU. The representative flow cytometry patterns of cell cycle distribution is shown in **(D)** and the statistical analysis is shown in **(E)**. The representative patterns of cell apoptosis are shown in (**F**, upper panel) and the statistical analysis is shown in (**F**, lower panel). **(G)** Overexpression of miR-130b in AGS cells and 5-FU caused DNA damage detected by comet assay. Representative images are shown in (**G**, left panel) and the mean ± SD for each condition shown in (**G**, right panel). **(H, I)** AGS cells were cotransfected with the CMPK1 CDS region or negative control vector together with 20 nM miR-NC or miR-130b. After 24 h, cells were harvested for western blot analysis **(H)** or reseeded for 5-FU sensitivity assay **(I)**. Data represent the mean ± SD, *P < 0.05; **P < 0.01, two-sided Student’s t test.

Although miR-130b overexpression decreased CMPK1 levels, we needed to examine whether miR-130b regulation of CMPK1 expression actually influences the 5-FU sensitivity of GC cells. Functional experiments with a specific mimic and inhibitor were used to identify the role of miR-130b in GC cell chemoresistance. As expected, miR-130b-transfected GC cells were more resistant to 5-FU treatment than controls (MGC-803 survival percent for 10μg/ml 5-FU treatment, P = 0.003; AGS survival percent for 20μg/ml 5-FU treatment, P = 0.012) (**Figure 4A** **).** As indicated above, we also selected AGS cells for the following study because of its relatively low miR-130b expression levels confirmed in real-time PCR results (**Supplementary Figure S3E**). This miR-130b-induced insensitivity to 5-FU was also confirmed in a clonogenic survival assay showing that the growth inhibition effect on AGS cell was absent (**Figure 4B** **).** Conversely, anti-miR-130b transfection enhanced CMPK1 expression and induced higher sensitivity to 5-FU in MGC-803 and AGS cells (**Figure 4C** **)**.

To determine whether the expression of miR-130b in the presence of 5-FU could affect cell cycle arrest, we performed flow cytometry analyses in AGS cell lines transfected with a miR-130b mimic or miR-NC. Consistent with low-expression of CMPK1, a significant increase in the percentage of cells in G1-phase, but a reduced proportion in S phase was found in miR-130b over-expressing cells (**Figures 4D, E** **)**. Furthermore, to investigate how miR-130b may affect the ability of 5-FU to induce apoptosis, AGS cells were treated with 5-FU and harvested for flow cytometry analysis. The results indicated that the apoptotic rates of AGS-miR-130b cells were significantly lower than that of AGS-miR-NC cells (P < 0.001) (**Figure 4F** **)**.

Since CMPK1 has been verified to be involved in mediating 5-FU-induced DNA damage, we examined the role of its upstream regulator-miR-130b in DNA damage by comet assay. AGS cells with ectopic overexpression of miR-130b had lower levels of CMPK1 protein and significantly lower DNA damage than control cells (**Figure 4G**, left panel). The quantified percentage of DNA in the tail was significantly different in cells treated by miR-NC plus 5-FU vs miR-130b plus 5-FU (P <0.001, right panel). In addition, MTT assay suggested that the effect of miR-130b on 5-FU sensitivity was fully rescued by overexpressing CMPK1 coding region sequence (without its 3′-UTR) (**Figures 4H, I** **)**. Taken together, these indicated that miR-130b-mediated sensitivity to 5-FU is primarily a result of CMPK1 expression suppression.

### MiR-130b Regulated CMPK1 Influences Chemoresistance Primarily in 5-FU Treatment But Not Cisplatin

**Figure 5:**
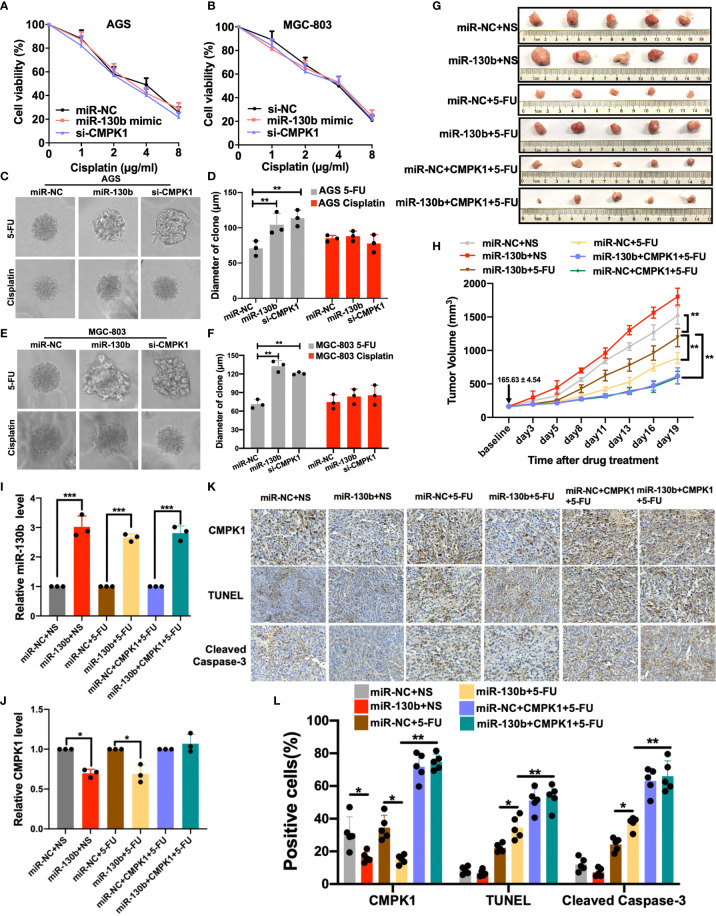
MiR-130b-mediated regulation of CMPK1 influences chemoresistance to 5-FU **(A, B)** AGS and MGC-803 cells were transfected with miR-NC, miR-130b mimic or si-CMPK1. After 48 h, cells were reseeded for cisplatin sensitivity detection by MTT assay. **(C, D)** The 3-D culture assay showed that 5-FU treatment, but not cisplatin, significantly impacted miR-130b/miR-NC/si-CMPK1 cell growth on martrigel matrix. AGS cell spheroids, morphological changes, and the diameter of clone is shown in **(C, D)**, MGC-803 cell spheroids are shown in **(E, F)**. **(G)** Representative images of orthotopic gastric cancer mouse model for 5-FU sensitivity in miR-NC, miR-130b, and CMPK1-treatment. The scale was millimeters. **(H)** Mean tumor volumes in mice treated with different combination of miR-NC, miR-130b, CMPK1 and 5-FU regimens. Expression level of miR-130b **(I)** and CMPK1 **(J)** in different xenograft mouse model by q-PCR analysis. **(K)** Tumor samples from control- and miR-130b-treated mice were sectioned and stained for CMPK1, TUNEL and Cleaved Caspase-3 by immunohistochemistry (IHC). **(L)** Quantification of CMPK1, TUNEL and Cleaved Caspase-3 positive cell level. Error bars, ± SD. (*P < 0.05, **P < 0.01, ***P < 0.001).

Since CMPK1 activated deoxycytidine analogs of 5-FU and sensitized GC cells to cytotoxicity, we wanted to determine whether the effect could extend to another GC first-line chemotherapy drugs cisplatin. AGS and MGC-803 cells were transfected with a standard concentration (20 nmol/L) of miR-130b/control/si-CMPK1 and subsequently treated with cisplatin. The MTT results (**Figures 5A, B** **)** and clone formation assay (**Supplementary Figures S4A, B** **)** showed no difference on cell viability. Furthermore, we performed flow cytometric analyses in AGS cell lines transfected with miR-130b/control/si-CMPK1 treated with cisplatin and found no difference between based on transfection status (**Supplementary Figures S4C, F**). These results imply that transfected with miR-130b/control/si-CMPK1 had no impact on cisplatin treatment (P > 0.05).

Interestingly, the 3-D spheroid culture assays showed that in 5-FU treatment group, the diameters of GC cell spheroids of treated by miR-130b/si-CMPK1 were significantly increased than control in mean diameters and numbers of cell clones (miR-NC *vs* miR-130b, MGC-803: P < 0.001; AGS: P < 0.001. miR-NC *vs* si-CMPK1, MGC-803: P = 0.001; AGS: P < 0.001) (**Figures 5C–F** **)**. Conversely, we found no impact on spheroid size in cisplatin treated cells when transfected with miR-130b/control/si-CMPK1 (**Figures 5C–F**). And clone formation assay demonstrated consistent results (**Supplementary Figures S5A–D** **).**

To further assess the ability of miR-130b/CMPK1 to induce 5-FU sensitivity, we tested the therapeutic efficacy of a miR-130b, CMPK1 and 5-FU combination in an established xenograft nude mouse model (see Supplementary Methods for details). For the 5-FU treatment model, following subcutaneous injection of AGS or CMPK1-CDS overexpressed AGS cells, mice were randomly distributed and assigned to the following treatment groups: 1) miR-130b/NC plus NS, 2) miR-130b/NC plus 5-FU. 3) miR-130b/NC plus CMPK1 plus 5-FU. As compared with the control miRNA group, tumors in the miR-130b group had statistically significantly more tumor burden based on tumor volume (P < 0.001) (**Figures 5G, H** **)**. While the addition of 5-FU led to decreased tumor volume, the combination of miR-130b plus 5-FU led to less reduction in tumor burden compared with miR-NC plus 5-FU (P <0.001) (**Figure 5H** **)**. Moreover, CMPK1-CDS overexpressed GC cells plus 5-FU exhibited the strongest tumor suppression effects regardless of combination of miR-130b or control, further suggested miR-130b on 5-FU sensitivity was fully rescued by overexpressing CMPK1 coding region sequence.

We further compared the miR-130b and CMPK1 expression in mice tumor tissues by Real-time quantitative PCR analysis. Transfection of miR-130b mimics significantly enhanced the miR-130b expression while reduced the CMPK1 expression (**Figures 5I, J** **)**. Meanwhile, xenograft mice models injected with CMPK1-CDS overexpressed cells resulted in a significant augmentation of CMPK1 expression whether combination of miR-130b or miR-NC (**Figure 5J** **).** We also conducted the immunohistochemical staining of CMPK1, TUNEL and Cleaved Caspase-3 in the xenograft mice tumors to determine whether systemic delivery of miR-130b affected the expression of CMPK1 and apoptosis. Representative sections stained for these markers are shown in revised **Figure 5K**. Compared with miR-NC, miR-130b treatment exhibited significantly lower level of CMPK1-positive and higher levels of TUNEL- and Cleaved Caspase-3-positive cells in 5-FU-treated mice tumors (P < 0.05; **Figure 5L**).

## Discussion

Development of chemoresistance is a major cause of treatment failure in GC patients treated with 5-FU. The origins of acquired drug-resistance can be stem from multiple mechanisms, but the efficiency of drug metabolism often affects chemotherapeutic efficacy. Previous studies have found that UMP/CMPK played a critical role in 5-FU phosphorylated activation (29, 30), while the mechanism of led to the 5-FU resistance remains unknown. Beyond the mutational processes that can affect the expression or activity of drug metabolism genes, epigenetic regulation resulting in gene silencing by miRNAs has always been known to deregulate drug-resistance-related functions (16, 19, 31, 32). In the present study, we first validated the clinical efficacy of the TCGA-defined sensitive subtype in 5-FU-based chemotherapy and identified the CMPK1 as the top DEGs. Lower CMPK1 expression was associated with worse response to 5-FU-based therapy and shorter survival in GC patients. Further, CMPK1 could be regulated by miR-130b *via* directly targeting the gene’s 3′-UTR, and attenuated 5-FU chemosensitivity *in vitro* and *in vivo*. However, we have not detected similar effects in cisplatin-treated cells, suggesting that miR-130b/CMPK1 axis may specifically regulate 5-FU metabolism. This study reports for the first time that overexpression of miR-130b attenuated cell chemosensitivity to 5-FU, possibly due to reduced drug activation *via* downregulation of genes involved in the phosphorylation of FUMP, including CMPK1, suggesting that miR-130b may contribute to chemotherapy resistance.

Previous clinical trials showed that regimens containing 5-FU improve survival in tumor, but that local failure and distant metastases still frequently occur (33, 34). CMPK1 is a member of nucleoside monophosphate kinase family and is highly homologous to adenylate kinase (35). A recent study showed that methylation inhibitors restored sensitivity to 5-FU after bolus administration, which is mediated by increased CMPK1 levels resulting in decreased clinical resistance to 5-FU due to decreased CMPK1 in colorectal cancer (29). Further, modulation of CMPK1 by overexpression or down-regulation had no impact on natural pyrimidine nucleotides and cell growth (30). In this study, down-regulating CMPK1 expression by siRNA led to a decrease in the formation of the 5-FU triphosphate metabolites, resulting in cellular resistance to 5-FU–based treatment.

Currently, our understanding of the biological functions of miR-130b in gastric cancer is still limited and sometimes inconsistent. Lai et al. reported that overexpression of miR-130b increased cell viability, reduced cell death and decreased expression of Bim *via* regulating of tumor suppressor RUNX3 (36). Zhang et al. found miR-130b delivered in M2 macrophage-derived extracellular vesicles promoted survival, migration, invasion, and angiogenesis of GC cells (37). A recent study showed that plasma miR-130b expression were associated with response to 5-FU/oxaliplatin treatment in metastatic colorectal cancer (mCRC) and upregulated in non-responders (38). However, other studies highlighted its implication in the cisplatin chemoresistance of lung and ovarian cancer (39, 40). These results, which conflict with our studies, may be attributed to varying functions of the multiple subtypes of miR-130b in different types of cancer. Interestingly, no published study has focused on 5-FU chemoresistance mediated by miR-130b in GC. To test the potential role of miR-130b in GC, we performed statistical analysis with microarray data and q-PCR, and showed that a high level of miR-130b expression was associated with reduced response to 5-FU-based therapy in GC patients. We further analyzed downregulated genes *via* mRNA microarray expression combined with worse prognosis and observed a decrease in CMPK1 levels. The 5-FU chemoresistance biomarker TYMS was also associated with miR-130b/CMPK1 expression. Subsequently, the role of miR-130b in mediating gastric cancer chemoresistance was confirmed by validating CMPK1 as a direct target of miR-130b *via* luciferase and western blot assays, and demonstrating that miR-130b could reduce 5-FU sensitivity and DNA damage.

In this study, the observation that the miR-130b-induced 5-FU cytotoxic attenuation was largely rescued by overexpressing CMPK1, suggests that CMPK1 is the key target for miR-130b-enhanced 5-FU inactivation and drug resistance. Elevated CMPK1 levels and enhanced sensitivity to 5-FU following miR-130b down-regulation may have important clinical relevance. Despite recent advances in genomic sequencing, effective molecular targeting drugs for GC have not yet been established; therefore, conventional chemotherapy including drugs such as cisplatin or 5-FU remains important for the treatment of GC (41).

Whereas the association of CMPK1/miR-130b with 5-FU sensitivity was seen in TCGA cohort, the dataset were retrospective analysis and sample size is still relatively small, thus, further validation in additional large prospective cohorts is important. Additionally, we validated the miR-130b functions associated with fluorouracil mainly in gastric cancer; further analyses of which effect in another gastrointestinal tumor will also be needed.

**Figure 6:**
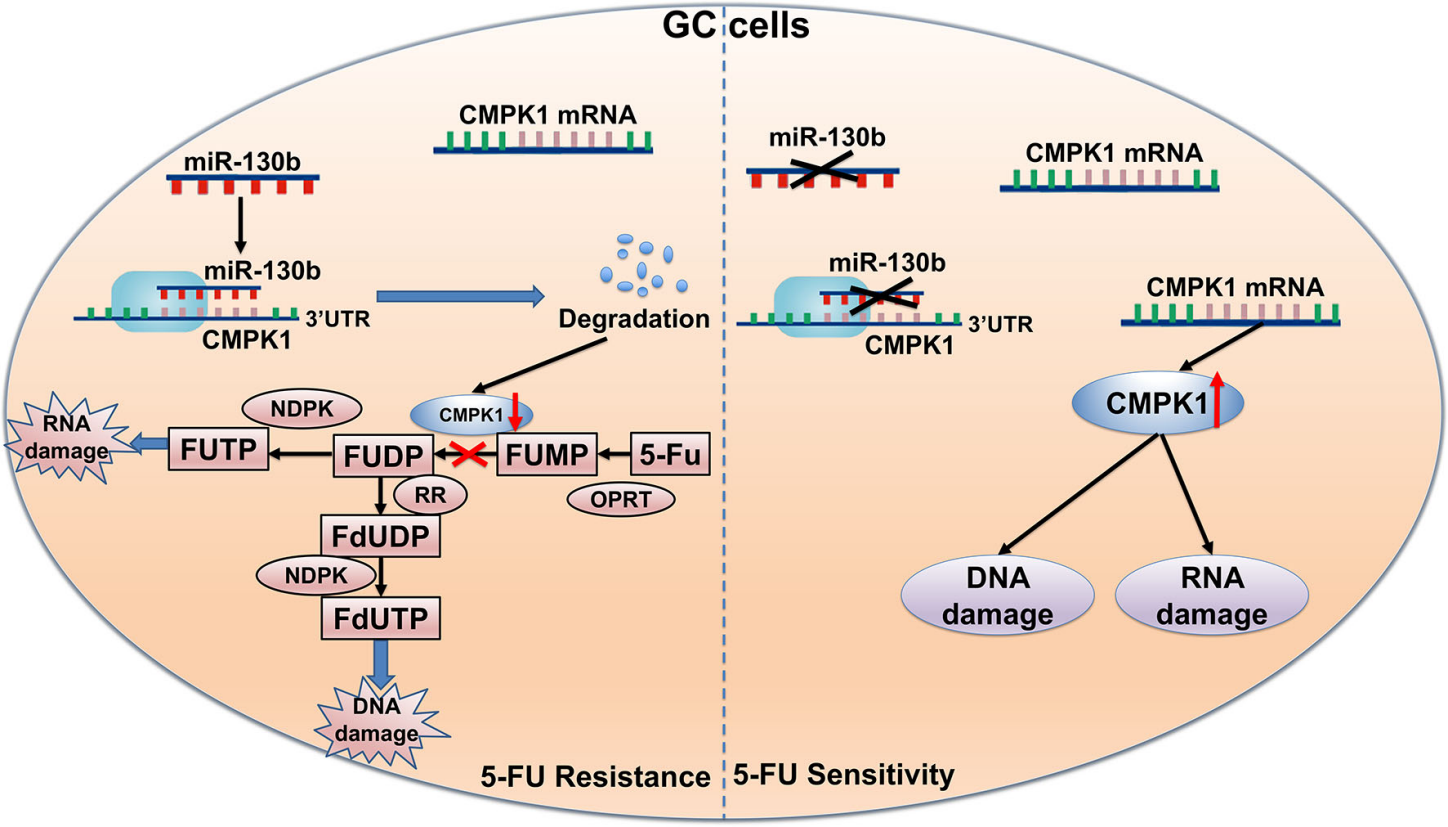
Schematic of the proposed molecular mechanism of miR-130b in GC affecting 5-FU metabolism. First, 5-FU is converted to FUMP *in vivo* by OPRT. MiR-130b directly targets CMPK1 expression and suppresses the phosphorylation of 5-FU FUMP to the diphosphate metabolites FUDP and FdUDP. As a result, miR-130b attenuates the response of gastric cancer cells to chemotherapy and impacts survival. FUMP, 5-fluorouridine-5′-monophosphate; FUDP, 5-fluorouridine-5′-diphosphate; FdUDP, 5-fluoro-2′-deoxyuridine-5′-diphosphate.

Development of chemoresistance is a persistent problem in gastric cancer patients, and establishing a novel strategy to overcome this is needed. The discovery of a miR-130b-CMPK1–Fluorouracil DNA damage axis supports the approaches that combining miR-130b inhibition with 5-FU agents may substantially benefit gastric cancer management (**Figure 6** **).** Our findings suggest that a miR-130b inhibitor combined with 5-FU chemotherapy may strengthen the chemosensitivity and provide a novel therapeutic method for treating GC patients. In addition, our findings suggest that miR-130b might be a valuable predictive biomarker for the chemotherapy response in GC patients, and provide a therapeutic drug target in the neoadjuvant chemotherapy setting.

## Data Availability Statement

The data sets presented in this study can be found in online repositories. The names of the repository/repositories and accession number(s) can be found in the article/**Supplementary Material**.

## Ethics Statement

The animal study was reviewed and approved by the medical ethics committee at Qinghai Provincial People’s Hospital.

## Author Contributions

JX, HC, and NH designed the project. JX, HC, and NH performed administrative, technical, or material support. HC and NH performed statistical analysis. HC and NH wrote the manuscript. HC and JX revised the paper. All authors contributed to the article and approved the submitted version.

## Conflict of Interest

The authors declare that the research was conducted in the absence of any commercial or financial relationships that could be construed as a potential conflict of interest.
