# Supplementary material for: CMPK1 Regulated by miR-130b Attenuates Response to 5-FU Treatment in Gastric Cancer
Source: Front Oncol. 2021 Mar 18;11:637470. doi: 10.3389/fonc.2021.637470 (PMC8013733; doi:10.3389/fonc.2021.637470)
Supplement: Supplementary file 1 [file DataSheet_1.docx]

Supplementary Material

# Supplementary methods

**RNA extraction Quantitative RT-PCR analysis**

Total RNAs were extracted using Trizol Reagent (Life technology, USA) in accordance with the manufacturer’s instructions. The expression of candidate microRNA was quantified by reverse-transcription polymerase chain reaction (RT-PCR) according to the Taqman microRNA assay protocol (Applied Biosystems, Carlsbad, CA). The microRNA cDNA was prepared from total RNA using the Taqman microRNA Reverse Transcription kit and microRNA-specific stem-loop primers in the RT reaction according to the manufacturer's instructions. Data were analyzed with SDS Relative Quantification Software version2.1 (Applied BioSystems), with the automatic Ct setting for assigning baseline and threshold. The amount of microRNA relative to U6 small nuclear RNA was determined using the ΔΔCt method, normalizing the results to the endogenous U6 small nuclear RNA expression level.

**Luciferase assay**

CMPK1 3′-UTR sequence was amplified from cDNA with the CMPK1 3′-UTR up primers SacI (5′-GAGCT'CGCTTCCTTTCATCAGGTATC-3′) and down primers XhoI (5′-CTCGAGCATCCAACATCACTGAATGG-3′). The PCR products were then subcloned to the pmirGLO dual-luciferase target expression vector (Promega, USA) as wild-type vector pmirCMPK1-3′-UTR-Wt (CMPK1-Wt). The mutant vector pmirCMPK1-3′-UTR-Mut (CMPK1-Mut) was obtained by site-directed mutagenesis using QuikChange® Site-Directed Mutagenesis Kit (Stratagene, USA). AGS and MGC-803 were seeded in a 24-well culture plate in triplicate and were cotransfected with 130b mimic and miR-NC followed by CMPK1-Wt or CMPK1-Mut using DharmaFECT Duo Transfection Reagent (Thermo, USA) according to the manufacture’s procedure. Luciferase activity was normalized to that of pRL-TK luciferase. The cells were collected at 24h post-transfection; luciferase activity was measured by a dualluciferase reporter assay kit (Promega, USA) and recorded by a GloMax 20/20 (Promega, USA).

**Cell-cycle analysis**

Cultured cells were seeded onto the 6-well plates at a density of 1 × 10^6^ cells per well and incubated overnight. After treated with or without 10 μg/ml 5-FU for 48 h, cells were collected and washed with PBS. Cell pellets were fixed in 70% cold ethanol overnight at -20°C. The fixed cells were washed in PBS and suspended in the Cell Cycle Reagent (Millipore, US) at 5 × 10^5^ cells/ml. The cells were incubated in the dark for 30 minutes at room temperature. The cell solutions were analyzed by a ﬂow cytometer, guava easyCyte™ (Millipore, US), to determine cell populations at different cell cycle phases. The DNA contents of the stained cells were analyzed using the Modft LT software (Verity Software House, US).

**Apoptosis** **analysis**

Cells (1 × 10^6^/well) were seeded in 6-well plates and treated with or without 20μg/ml 5-Fu. After incubation for 48 h, total cells were harvested and washed twice with cold PBS. The cells were resuspended in the Nexin Reagent (Millipore, US) with added 1% FBS at 2.5 × 10^5^ cells/ml and then incubated in the dark for 20 minutes at room temperature. The cell solutions were analyzed by a ﬂow cytometer, Guava™ easyCyte (Millipore, US), to determine cell populations at different apoptosis phases.

**Western Blot Analysis**

The protocol started with uploading 30 μg proteins from the whole cell lysate in each sample onto a 12% PAGE gel. After electrophoresis and gel transferring, the membrane was blocked with 5% non-fat milk in 1xTris-buffered saline (pH 7.4) containing 0.05% Tween-20, and then probed with primary antibodies at concentrations of 1:2000 for β-actin (Santa Cruz Biotechnology, US), 1:1000 for CMPK1 (Abcam, US) at 4°C overnight. The membrane was incubated with HRP-conjugated secondary antibody Rabbit Anti-Mouse (1:2000) (Santa Cruz, USA) for one hour at room temperature. The detected proteins were visualized using the Visualizer Western Blot Detection Kit (Millipore, US). Detection was performed by C-DiGit Chemiluminescent Western Blot Scanner (LI-COR, US).

**Comet assay**

Comet assays were performed per the manufacturer’s instructions (Trevigen, Gaithersburg, MD). Proliferating AGS cells were transfected with miR-NC, miR-130b mimic and si-CMPK1. Twenty-four hours later, transfected cells were treated with 20μg/ml 5-FU for 48 hours, and then analyzed by single-cell gel electrophoresis. Briefly, cells were collected and resuspended in ice-cold PBS at 1×10^5^ cells/ml, mixed with low-melt agarose (1:10 ratio), and spread onto frosted glass slides. After the agarose solidified in 4℃ for 30 minutes, the slides were successively placed in lysis and alkaline solutions (Trevigen). Slides were then subjected to electrophoresis (1 V per cm of distance between electrodes) for 20 minutes in 1× TBE buffer, and cells were fixed with 70% (v/v) ethanol and stained with DAPI. DNA damage was quantified for 100 cells for each experimental condition by determining the tail moment, a function of both the tail length and intensity of DNA in the tail relative to the total DNA, using the Comet Score software (CASP Lab). Gray level images were acquired under immunoﬂuorescence microscopy. Differences between groups were analyzed by the Student’s t-test.

**Xenograft mice models**

AGS blank cells or CMPK1-CDS-overexpressed AGS cells were trypsinized, washed, resuspended in Hanks balanced salt solution (Gibco, Carlsbad, CA) and injected subcutaneously (5×10^6^ cells per animal) into the flank of each nude mice (Balb/c nu, female, 4 weeks old). Seven days after tumor cell injection, the tumor-bearing mice were randomized into 6 groups (n=5 in each group) and treated as follows: miR-NC+NS, miR-130b+NS, miR-NC+5-FU, miR-130b+5-FU, CMPK1+miR-NC+5-FU, and CMPK1+miR-130b+5-FU. The microRNAs incorporated with vivo-JetPEI (Polyplus, Berkeley, CA) transfection agents were formulated according to the manufacturer's instructions with a final N/P ratio of 7. Briefly, 1 nmol of microRNA and 1.87 µl of JetPEI were each diluted to 50 µl with 5% glucose. The two solutions were then mixed and incubated for 15 min at room temperature. The entire mixture (100 µl) was injected subcutaneously into each BALB/C nude mouse. All groups received twice-weekly miRNA treatments for three to six weeks. 5-FU (20 mg/kg) was administered triple-weekly for four to six weeks via the intraperitoneal route. Tumors were measured by using a caliper and were weighted daily. The tumor volume was calculated by using the modified ellipsoid formula: (large diameter) × (small diameter)^2^/2. When treatment was completed, mice were killed and their tumors harvested. Tumor weights, numbers, and locations were recorded. All animal work procedures were approved by the Ethics Committee of the Qinghai Provincial People's Hospital.

# Supplementary Figures and Tables

## Supplementary Figures


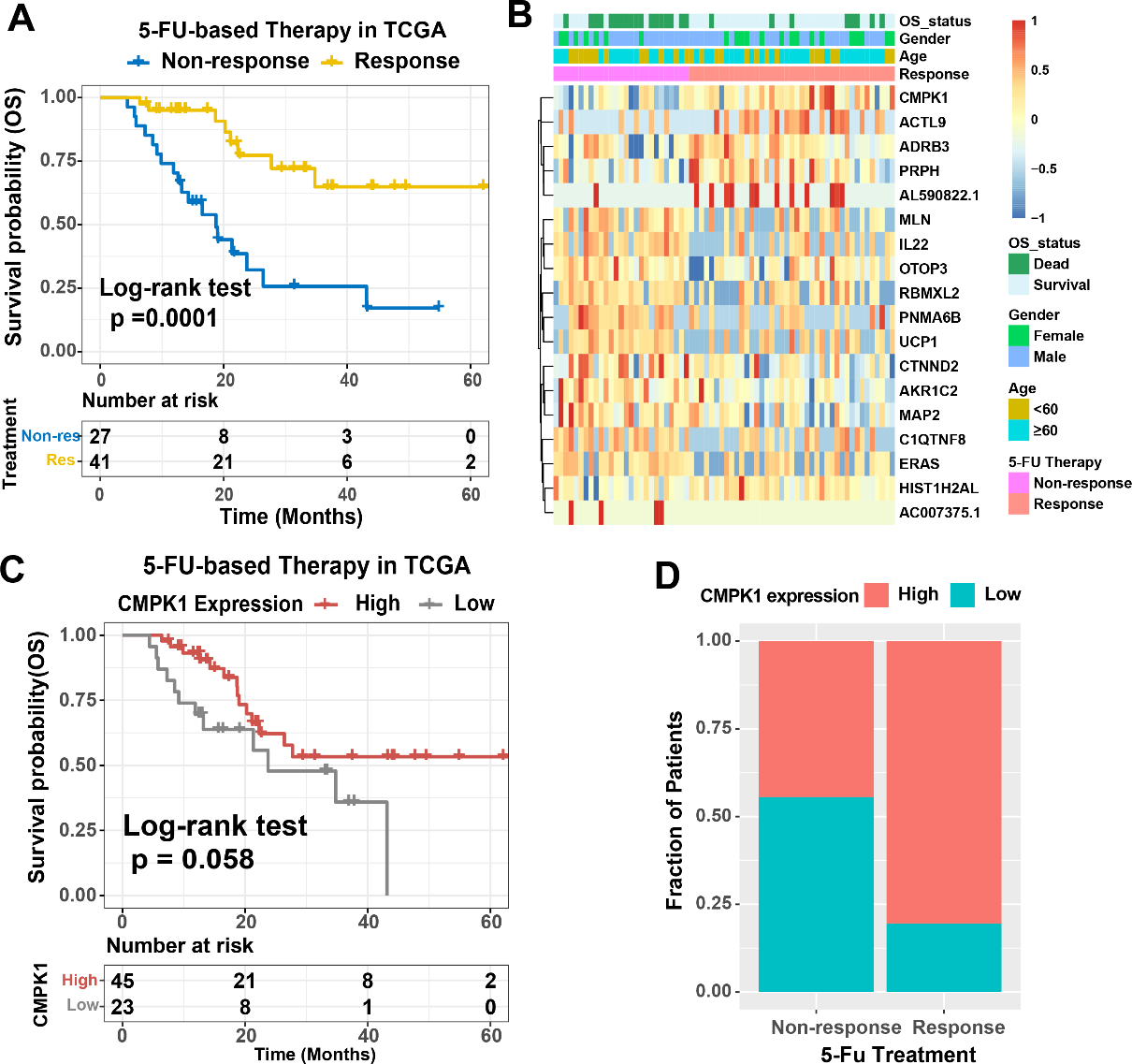


**Figure S1. Molecular marker associated with 5-FU treatment response in GC patients.** (A) Kaplan-Meier overall survival curves of 68 GC patients received 5-FU therapy with non-response (blue line) or response (yellow line). (B) Heatmap of the representative DEGs in different chemotherapy response subgroup. Clinical features of survival status, age, gender was displayed in top panel. (C) Kaplan-Meier overall survival curves of 68 GC patients received 5-FU therapy with different CMPK1 expression status. CMPK1 were stratified into low and high expression according to the lower tertile value. (D) Association of CMPK1 expression with 5-FU treatment response status.


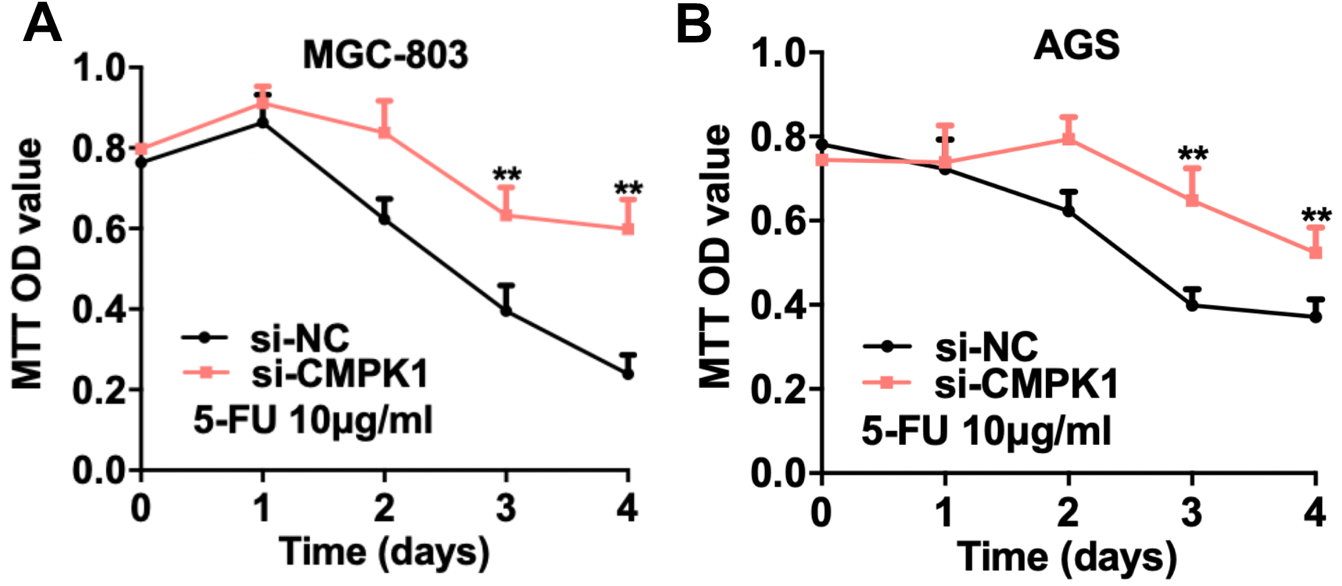


**Figure S2.** **Identification of cytotoxicity duration time of 5-FU treatment in GC cell.** (A-B) MGC-803 (A) and AGS (B) cells transfected with either si-NC or si-CMPK1 and cells were reseeded for 5-FU sensitivity detection in 24h, 48h, 72h and 96h using an MTT assay.


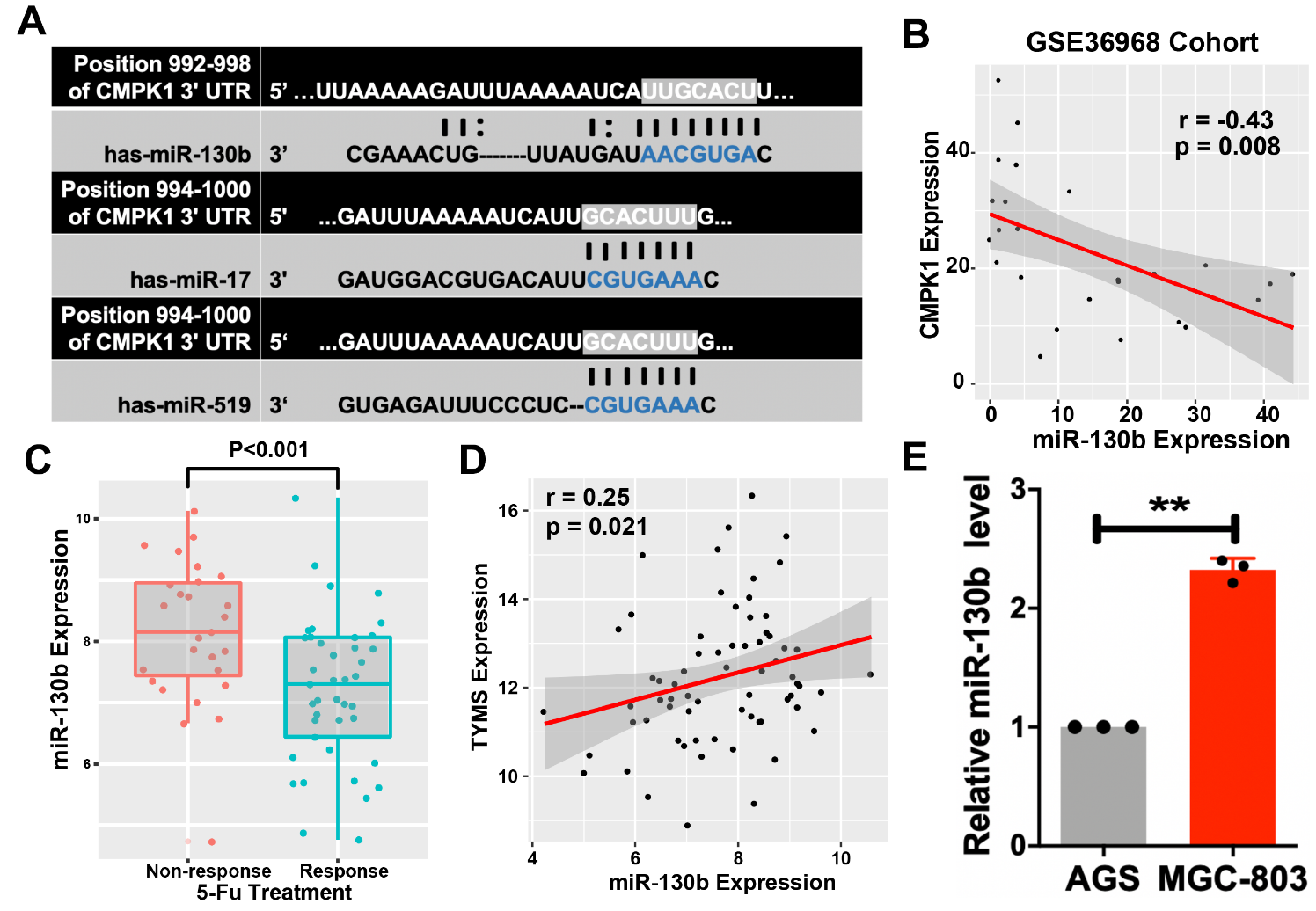


**Figure S3.** **Potential CMPK1 upstream regulative miRNAs in GC.** (A) The simulated binding sites of miRNAs with CMPK1 3’UTR region in TargetScan website. (B) Association of CMPK1 with miR-130b expression was validated in an independent cohort (GSE36968; r = -0.43, P = 0.008). (C) Higher expression of miR-130b was observed in 5-FU treatment non-response subgroup. (D) MiR-130b expression was positively correlated with 5-FU chemoresistance biomarker TYMS in TCGA cohort. (E) Relative expression levels of miR-130b in MGC-803 and AGS cell lines. The level of miR-130b in AGS was taken as 1.


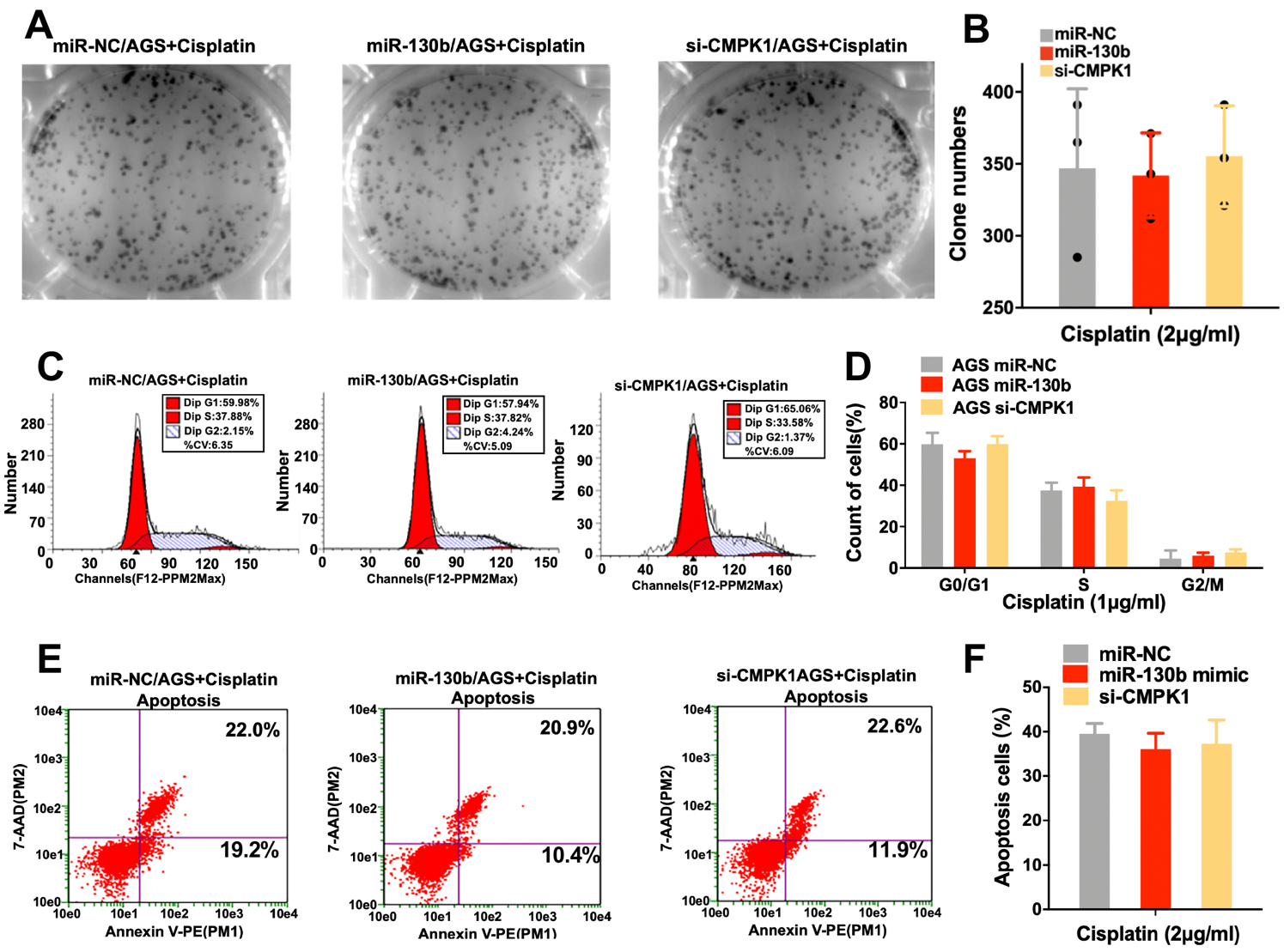


**Figure S4.** **AGS cells were transfected with miR-NC, miR-130b mimic or si-CMPK1, and cells were reseeded for cisplatin sensitivity assay.** (A) clonogenic cell-survival assay were performed to detect the impact of miR-130b and CMPK1 on cisplatin chemosensitivity. (B) Bar chart displayed the statistical results and indicated that there were no significant differences between control and miR-130b/si-CMPK1 on cisplatin. (C-F) Cell-cycle and cell apoptosis analysis were measured by ﬂow cytometry to determine the impact of CMPK1 and miR-130b on chemosensitivity to cisplatin. Cell cycle representative patterns were shown in (C), and the statistical results indicated that there were no significant differences between control and miR-130b/si-CMPK1 on cisplatin sensitivity (D). Representative patterns of cell apoptosis were shown in (E), and the statistical results indicated that there were no differences between control and miR-130b/si-CMPK1 on cisplatin induced cell apoptosis (F).


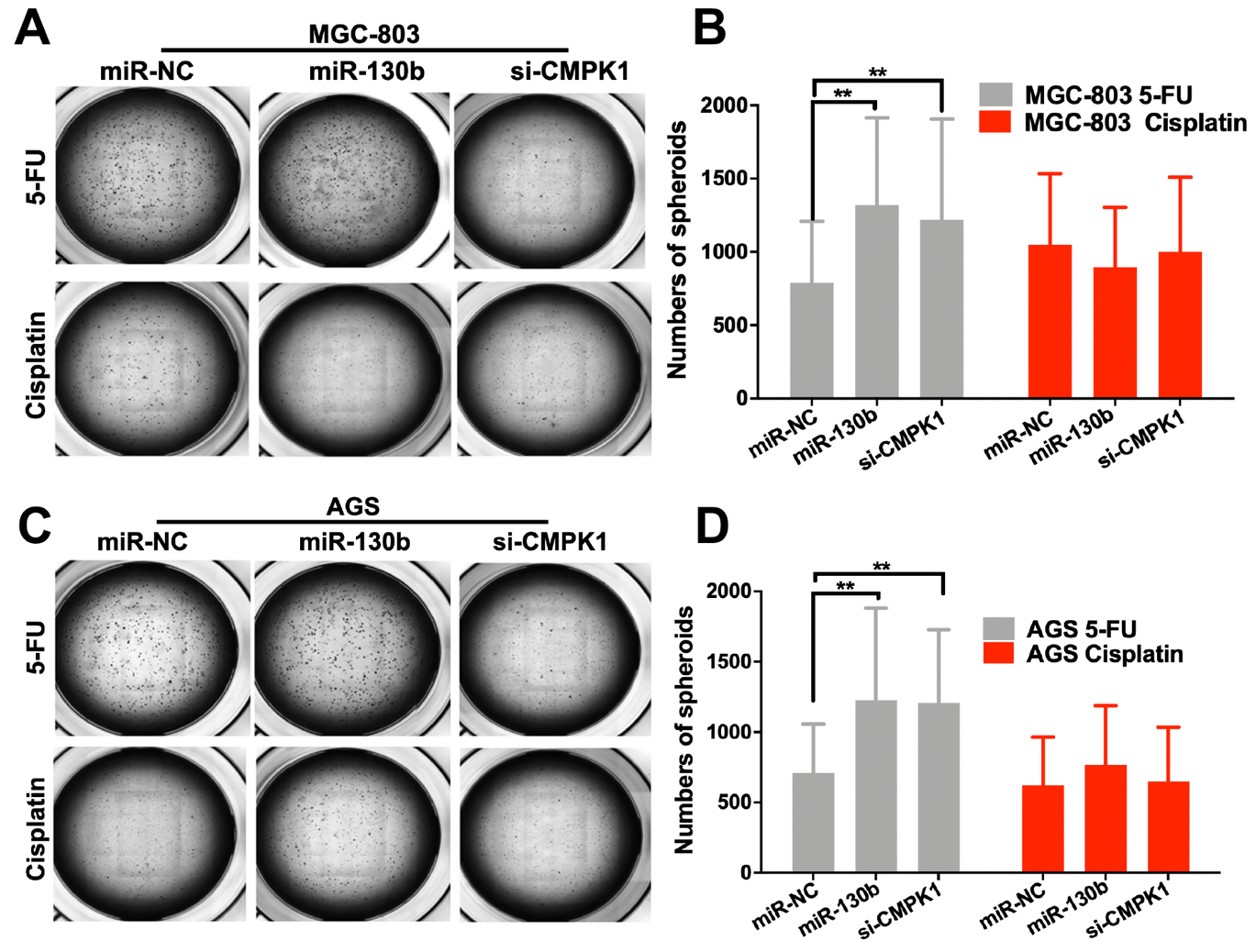


**Figure S5. 3-D culture assay showed that 5-FU treatment but not cisplatin significantly impacts transfected miR-130b/si-CMPK1 cells growth on martrigel matrix (A, C). The number changes of AGS and MGC-803 spheroids were shown in (B, D).**

**Table S1. The clinical characteristics of gastric cancer patients who received 5-FU-based chemotherapy in TCGA dataset.**
