# Supplementary figures and images for: CMPK1 Regulated by miR-130b Attenuates Response to 5-FU Treatment in Gastric Cancer
Source: Front Oncol. 2021 Mar 18;11:637470. doi: 10.3389/fonc.2021.637470 (PMC8013733; doi:10.3389/fonc.2021.637470)

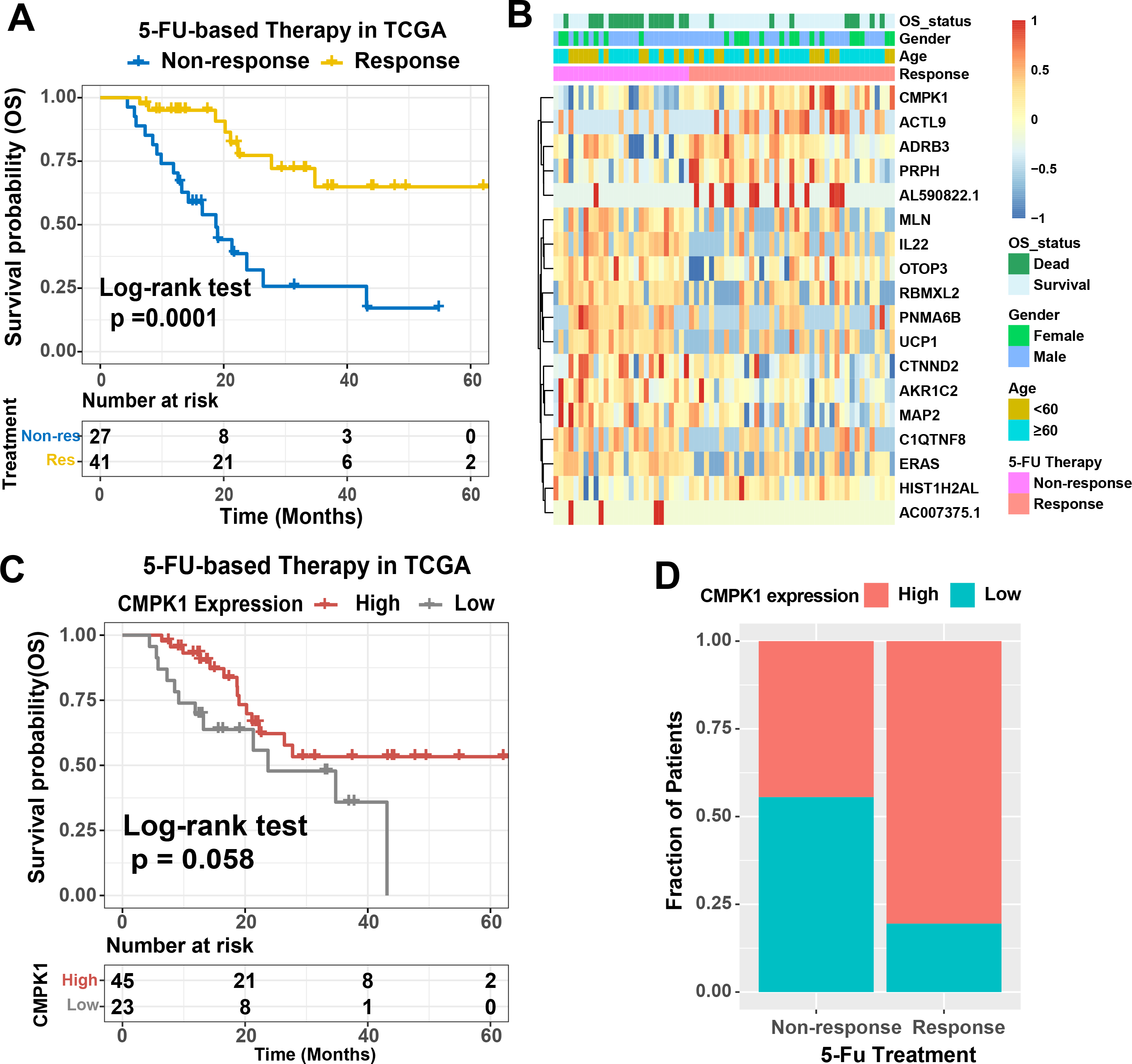

Supplement: Supplementary file 2 [file Image_1.tiff]

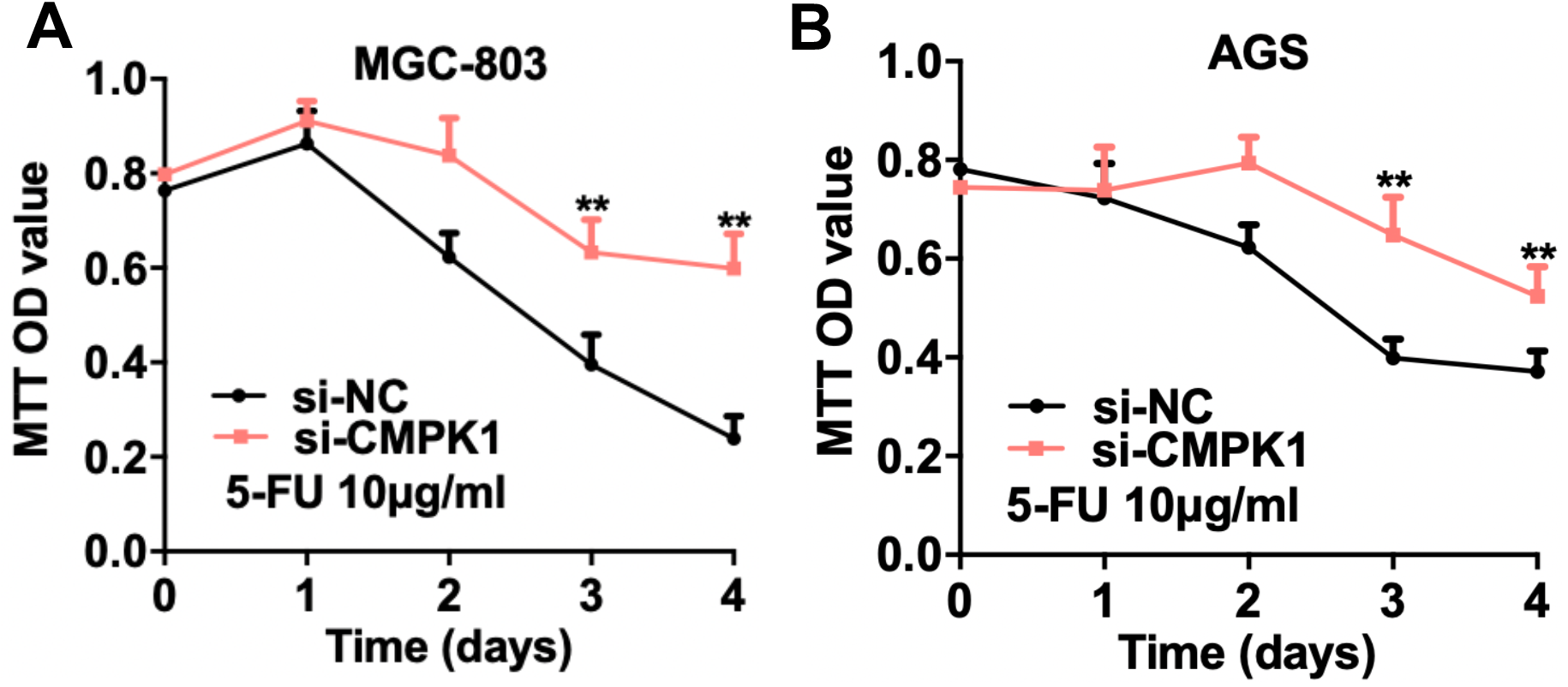

Supplement: Supplementary file 3 [file Image_2.tiff]

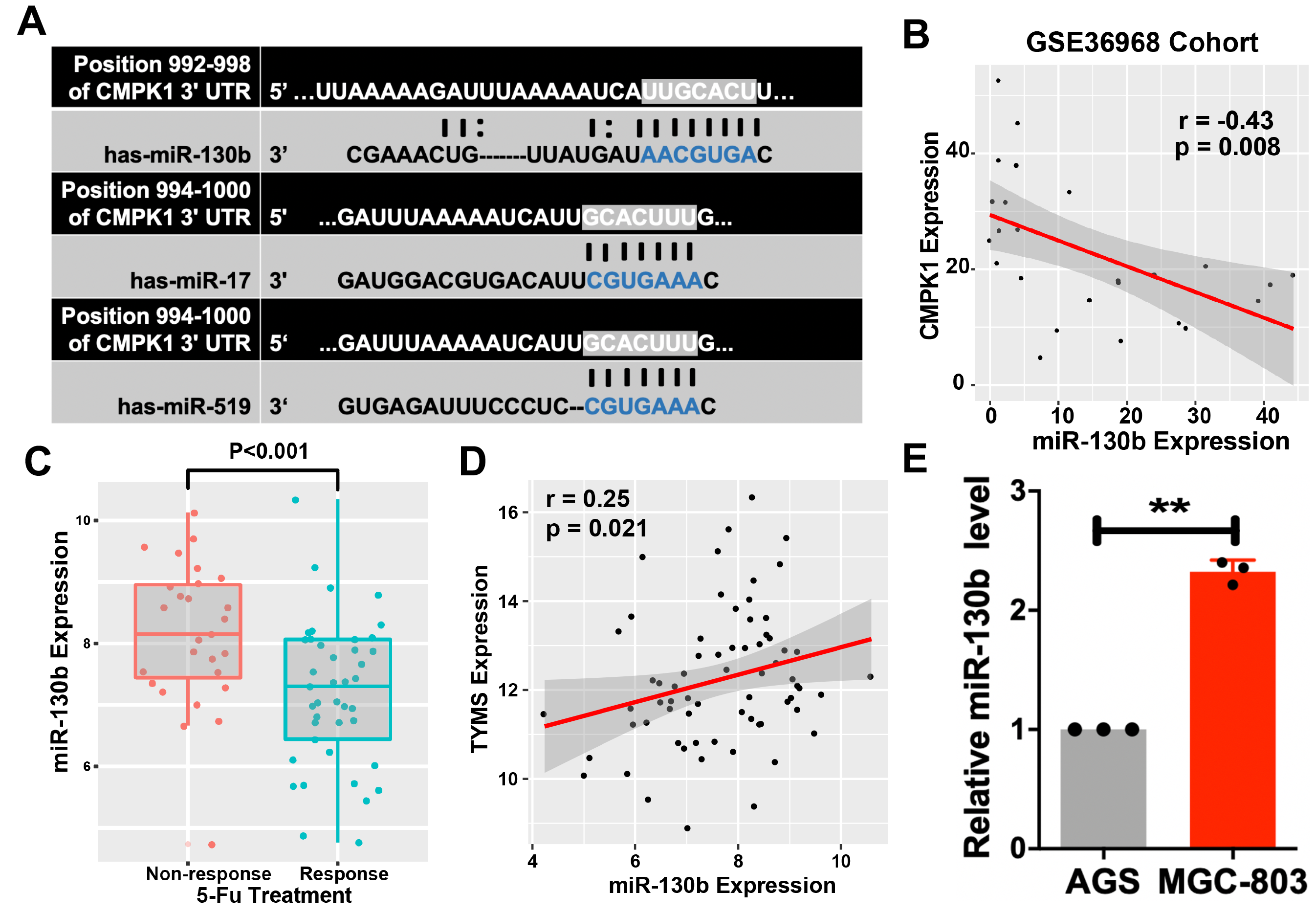

Supplement: Supplementary file 4 [file Image_3.tiff]

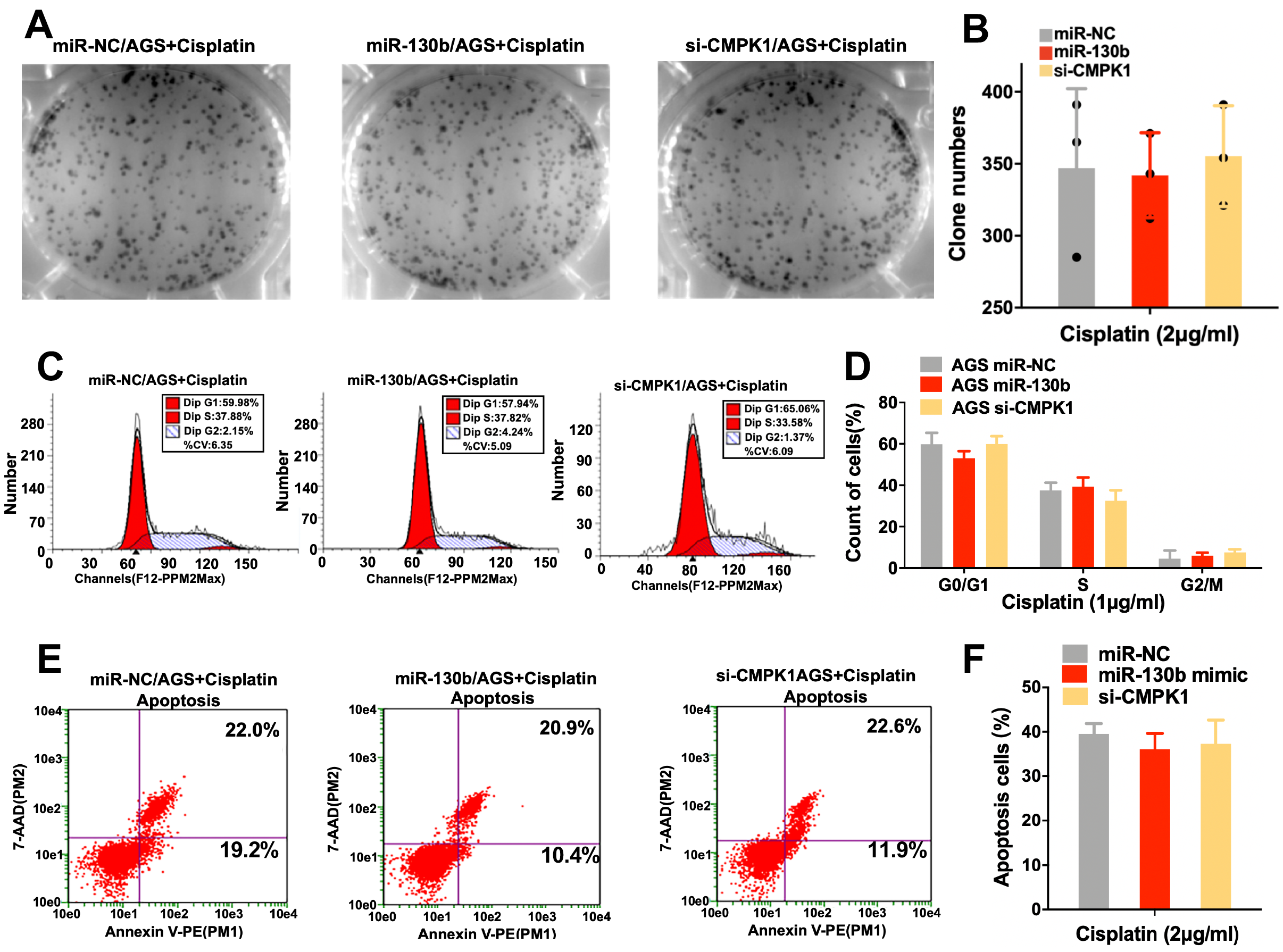

Supplement: Supplementary file 5 [file Image_4.tiff]

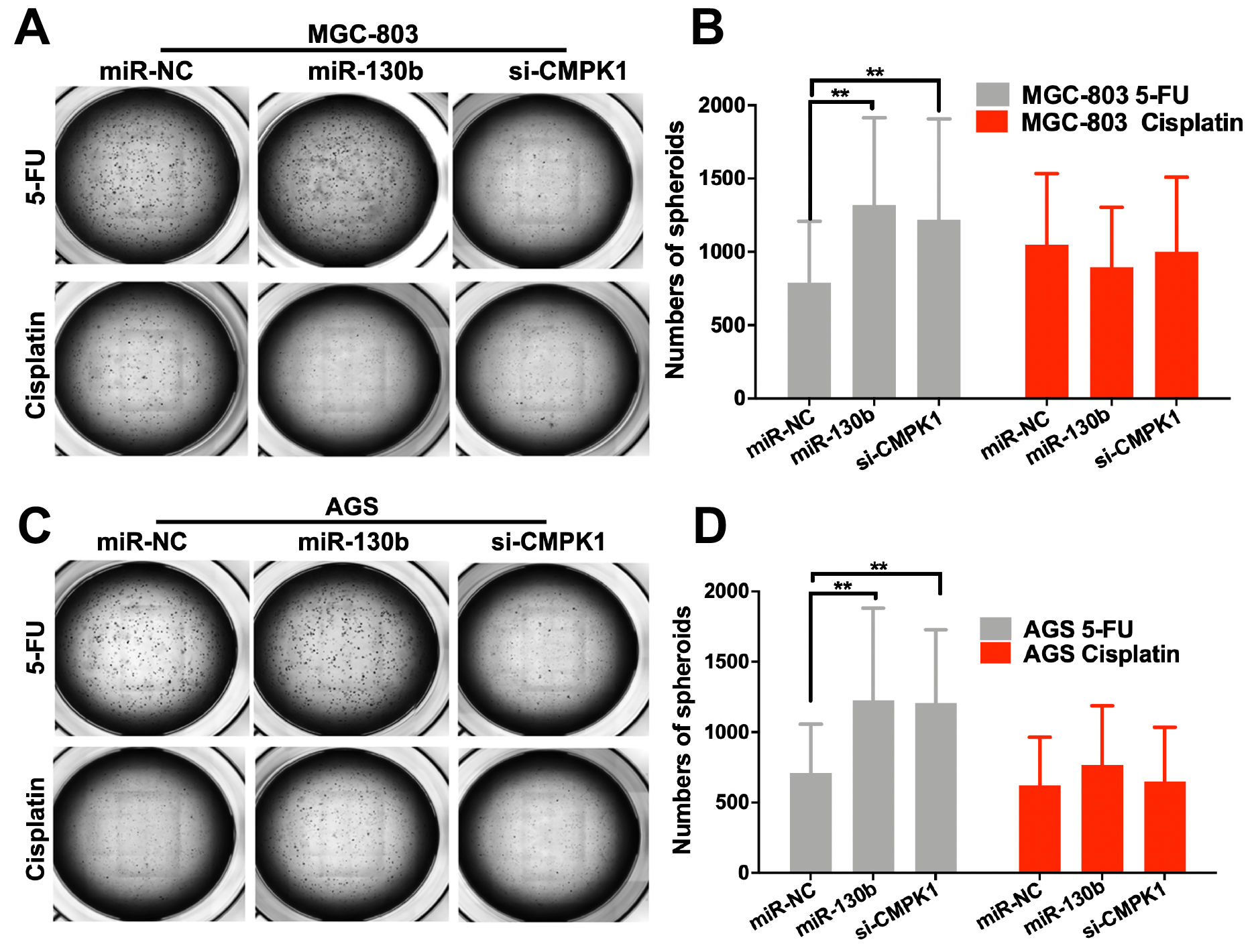

Supplement: Supplementary file 6 [file Image_5.tiff]
